# A Cough-Based Algorithm for Automatic Diagnosis of Pertussis

**DOI:** 10.1371/journal.pone.0162128

**Published:** 2016-09-01

**Authors:** Renard Xaviero Adhi Pramono, Syed Anas Imtiaz, Esther Rodriguez-Villegas

**Affiliations:** Department of Electrical and Electronic Engineering, Imperial College London, London, United Kingdom; Universidad Nacional de la Plata, ARGENTINA

## Abstract

Pertussis is a contagious respiratory disease which mainly affects young children and can be fatal if left untreated. The World Health Organization estimates 16 million pertussis cases annually worldwide resulting in over 200,000 deaths. It is prevalent mainly in developing countries where it is difficult to diagnose due to the lack of healthcare facilities and medical professionals. Hence, a low-cost, quick and easily accessible solution is needed to provide pertussis diagnosis in such areas to contain an outbreak. In this paper we present an algorithm for automated diagnosis of pertussis using audio signals by analyzing cough and whoop sounds. The algorithm consists of three main blocks to perform automatic cough detection, cough classification and whooping sound detection. Each of these extract relevant features from the audio signal and subsequently classify them using a logistic regression model. The output from these blocks is collated to provide a pertussis likelihood diagnosis. The performance of the proposed algorithm is evaluated using audio recordings from 38 patients. The algorithm is able to diagnose all pertussis successfully from all audio recordings without any false diagnosis. It can also automatically detect individual cough sounds with 92% accuracy and PPV of 97%. The low complexity of the proposed algorithm coupled with its high accuracy demonstrates that it can be readily deployed using smartphones and can be extremely useful for quick identification or early screening of pertussis and for infection outbreaks control.

## Introduction

Pertussis, also called *whooping cough*, is a contagious respiratory disease caused by *Bordetella pertussis* bacteria in lungs and airways [1]. Its early symptoms include persistent dry coughs that progress into intense spells of coughing. This is usually, but not always, followed by a whooping sound due to the patient gasping for air. It mainly affects infants and young children and can be fatal if left untreated. The latest World Health Organization official report on the disease (2008) estimated 16 million cases of pertussis annually worldwide resulting in approximately 200,000 deaths [2]. Estimates from Public Health Agency of Canada report an even higher prevalence with up to 40 million cases each year resulting in 400,000 deaths [3]. Further, about 95% of the pertussis cases have occured in developing countries where pertussis is considered to be major cause of infant deaths [2].

A trained doctor can confirm pertussis diagnosis, in mostly unvaccinated cases, by listening to the cough sounds and asking about other symptoms. The gold standard, however, is a culture test performed by collecting nasopharyngeal specimens. Alternative to this are the polymerase chain reaction (PCR) test, and blood analysis with serology. However, all these laboratory tests are expensive, time-consuming and may not be available, particularly in rural areas and developing countries. This can hinder effective and timely treatment of patients and risks worsening their condition as well as further spreading of the infection to others.

A low-cost, quick and easily accessible solution is needed to provide pertussis diagnosis to people in developing nations where its prevalence, and mortality rate due to pertussis, is highest. Such a system needs to be fully automated, user-friendly, and highly accurate so that there are no barriers to its adoption and deployment. With the smartphone usage steadily rising in developing countries [4], this serves as an ideal platform on which such an automated system can be developed. This paper proposes a complete automatic pertussis diagnosis algorithm based on automatic segmentation and classification of cough and whoop sounds. When implemented on an embedded device or a smartphone, it can analyze audio signals obtained from the built-in microphone and provide prompt diagnostic result. This ability to provide pertussis diagnosis by processing audio signals on a smartphone can be extremely helpful to deliver timely and efficient treatment to places and people with limited or no access to healthcare.

In this paper, we propose a pertussis identification algorithm that is able to automatically segment individual cough and whoop sounds and subsequently classify them and present a pertussis diagnosis. The aim is to develop an algorithm using little computational resources to allow the algorithm to be deployed on low–cost smartphones particularly in areas where healthcare services are substandard.

## Review of Cough Detection and Classification Algorithms

Cough detection is an active research area in which several researchers have proposed methods for identifying cough sounds from audio recordings. These methods can be divided into three main categories: 1) automatic cough detection and segmentation (without classification), 2) automatic classification of coughs that are already detected, and 3) diagnosis of an illness based on the cough sound and type.

For automatic cough segmentation, Martinek et al. [5] extracted several time, frequency and entropy features and used a decision tree to discriminate between voluntary cough sounds and speech. They used data from 20 subjects, with 46 coughs from each subject, and reported median sensitivity and specificity values of 100% and 95% respectively. However, their method is subject–dependent since the subjects are required to cough at the beginning of each recording in order to obtain individual cough signal patterns. Barry et al. [6] used linear predictive coding (LPC) coefficients with a probabilistic neural network (PNN) classifier to create an automatic cough counting tool called Hull Automatic Cough Counter (HACC). This successfully discriminated between cough and non–cough events from 33 subjects with a sensitivity of 80% and specificity of 96%. Tracey et al. [7] developed an algorithm for cough detection to monitor patient recovery from tuberculosis. They extracted MFCCs from the audio signals of 10 test subjects. These were used to detect coughs with a combination of artificial neural network (ANN) and support vector machine (SVM) classifiers achieving an overall sensitivity of 81%. In both these methods the total number of coughs in the dataset were not reported. Swarnkar et al. [8] used other spectral features such as formant frequencies, kurtosis, and B–score together with MFCC features for cough detection. These were fed into a neural network resulting in a sensitivity of 93% and a specificity of 94% for a test dataset consisting of 342 coughs from 3 subjects only. Amrulloh et al. [9] used ANN classification to develop a cough detector using a non–contact recording system for pediatric wards achieving sensitivity and specificity values of 93% and 98% respectively using over 1400 cough sounds from 14 subjects. Matos et al. [10] extracted thirteen MFCCs which were classified using a Hidden Markov Model (HMM). Their test dataset consisted of 2155 coughs from 9 subjects and their method resulted in 82% sensitivity for cough detection. The total number of false detections was not reported, however, the average false positives per hour was 7 with a high variance between subjects. Liu et al. [11] used Gammatone Cepstral Coefficient (GMCC) features with SVM classification of 903 coughs from 4 subjects resulting in sensitivity and specificity of 91% and 95% respectively. Lucio et al. [12] extracted 79 MFCC and Fast Fourier Transform (FFT) coefficients and used k-Nearest Neighbor (kNN) for classification. From a dataset acquired from 50 individuals, their algorithm achieved sensitivity of 87% in classifying 411 cough sounds with specificity of 84%. Larson et al. [13] presented a method of cough detection using the built-in microphone of a mobile phone for data collection. Their algorithm, which was not implemented on the phone, used random forest classification with a maximum for 500 decision trees achieving 92% sensitivity on over 2500 cough sounds from 17 subjects. All of these methods aim to identify cough segments from audio recordings without the ability to classify them into specific cough types.

Classification of cough sounds is helpful in identifying the underlying cause of coughs so that the right treatment can be offered to the patients. Several algorithms for automatic cough classification have been published to identify various cough types but all of them rely on manual segmentation of cough sounds before automatic classification can be performed. Chatrzarrin et al. [14] studied the different phases of dry and wet coughs and found the second phase of dry coughs to have lower energy compared to wet coughs. They also noted that, during this phase, most of the signal power is contained between 0-750 Hz in case of wet coughs and 1500-2250 Hz in case of dry coughs. Using a simple thresholding method, they successfully identified 14 wet and dry coughs with 100% accuracy. Swarnkar et al. [15] used a Logistic Regression Model (LRM) based classifier to discriminate between dry and wet coughs from pediatric patients with different respiratory illnesses. They used several features including B–score, non-gaussianity, formant frequencies, kurtosis, zero crossing rate and MFCCs. For a test database with 117 coughs from 18 subjects, they reported sensitivities of 84% and 76% for detecting wet and dry coughs respectively. Kosasih et al. [16] developed an algorithm for automatic diagnosis of childhood pneumonia by assessing cough sounds and crackles. They used MFCCs, non–gaussianity index and wavelet features with a LRM classifier to differentiate between pneumonia and non–pneumonia cough sounds. Their method achieved sensitivity and specificity of 81% and 50% respectively for a total of 375 cough samples from 25 subjects. Specific to pertussis coughs, Parker et al. [17] studied the performance of three different classifiers for their classification. They used audio files of pertussis cough sounds available on the internet to create a dataset consisting of 16 non-pertussis cough signals and 31 pertussis cough signals. From this data, the cough sounds were then manually isolated and divided into three parts for which 13 MFCC features and the energy level were extracted. These features were subsequently classified using an ANN, a random forest classifier and a kNN classifier. For each of these classifiers the false positive error was 7%, 12% and 25% respectively while the false negative error was 8%, 0% and 0% respectively.

From the review above, it can be concluded that while there are algorithms for cough detection and specific cough classification, none of them perform fully automatic cough detection *and* classification. Further, there has been only one algorithm reported for pertussis cough classification which also relies on manual segmentation of cough sounds prior to classification. Additionally, most of the high-performing cough detectors use complex classifiers making them unsuitable for use resource-constrained devices.

## Material and Methods

A typical episode of whooping cough involves intense coughing, usually but not always, followed by the characteristic whooping sound. Hence, in order to identify a pertussis case, two main sounds are helpful: cough sound and the whooping sound. While whooping sound is quite specific to pertussis, cough sounds have a lot of variance depending on the cause of cough. Hence, it is important to distinguish between the different cough types after a cough sound gets detected.

**Fig 1:**
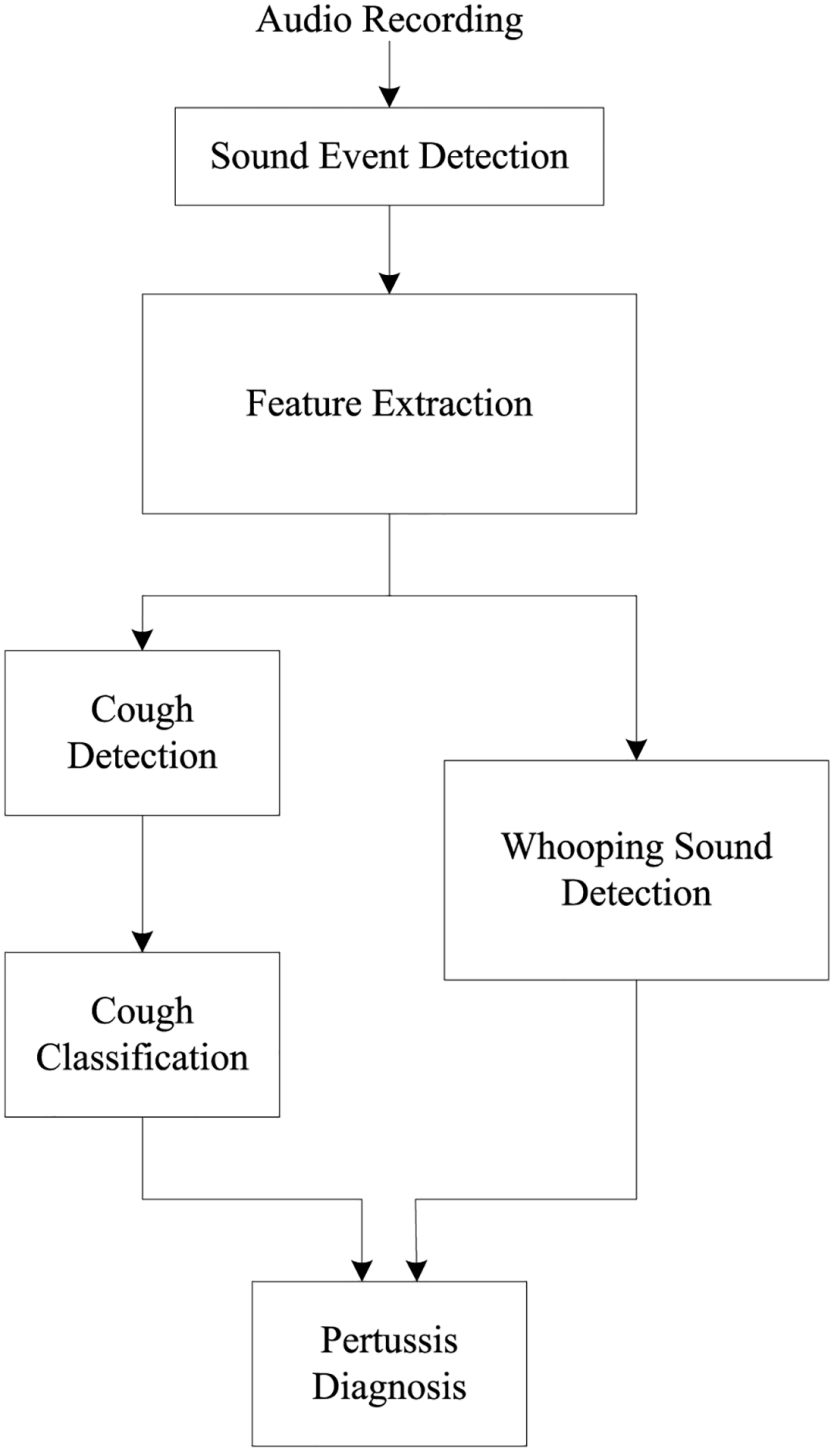
Block diagram of the automatic pertussis identification algorithm.

The pertussis diagnosis algorithm proposed in this paper, following the aforementioned approach, is shown in Fig 1. For the development of this algorithm, a total of 38 different audio recordings were acquired from publicly available sources with duration between 10–169 seconds and an average of 48.7 seconds per recording. (Table 1). These include 20 recordings of patients with pertussis cough, 11 with croup and other types of cough, and 7 of cough containing wheezing sounds corresponding to other diseases such as bronchiolitis and asthma. Of the 38 recordings, 14 are from infants aged 0-2 years, 18 from children aged 3-18 years and 6 from adults aged of over 19 years in age. Information about the exact laboratory test used to obtain the diagnosis for these recordings is, unfortunately, not available. Prior to any processing, all recordings were resampled to a frequency of 16000 Hz. This is because all the required information is contained below 8000 Hz, which is half of the new sampling rate. The audio signals were then divided into frames of 320 ms for processing with a 50% overlap between subsequent frames.

**Table 1:** List of data sources for cough sounds.

No	Source Link	Type	Age Group	Length (s)
TR1	https://www.youtube.com/watch?v=l-sNgKgAucI	Pertussis	Adult	32
TR2	https://workspace.imperial.ac.uk/rodriguez-villegas-lab/Public/whooping-cough/TR2.mp3	Pertussis	Infant	10
TR3	https://www.youtube.com/watch?v=TIV460AQUWk	Pertussis	Infant	139
TR4	https://www.youtube.com/watch?v=wuvn-vp5InE	Pertussis	Infant	10
TR5	www.whoopingcough.net/cough-child-muchwhooping.wav	Pertussis	Child	26
TR6	www.whoopingcough.net/paroxysm.wav	Pertussis	Child	35
TR7	https://www.youtube.com/watch?v=31tnXPlhA7w	Pertussis	Adult	56
TR8	https://www.youtube.com/watch?v=FIsQjsUJSiM	Pertussis	Adult	113
TR9	https://www.youtube.com/watch?v=Rmlo2to0ogs	Pertussis	Child	72
TR10	http://streaming.cdc.gov/vod.php?id=7ffe0c683b0dc2765090991b8f8018c920120904104432647	Pertussis	Child	15
TR11	http://www.youtube.com/watch?v=xwOfOgY8Ye8	Croup	Infant	123
TR12	http://www.youtube.com/watch?v=ID5KlHVJ91M	Croup	Child	16
TR13	http://www.youtube.com/watch?v=Qbn1Zw5CTbA	Croup	Infant	169
TR14	https://www.youtube.com/watch?v=Ro7HfT8oM8k	Unknown NP	Child	13
TR15	https://www.youtube.com/watch?v=8HWwSi1h0pw	Unknown NP	Child	20
TR16	https://www.youtube.com/watch?v=pAHDqQRDPCk	Bronchiolitis	Infant	73
TR17	https://www.youtube.com/watch?v=RFwr_zbgJII	Bronchiolitis	Infant	61
TE1	https://www.youtube.com/watch?v=AIVt3e5EVtc	Pertussis	Child	86
TE2	https://www.youtube.com/watch?v=KZV4IAHbC48	Pertussis	Child	51
TE3	www.whoopingcough.net/whoop-child-slightwhoop.wav	Pertussis	Child	16
TE4	www.whoopingcough.net/wc-adult.wav	Pertussis	Adult	21
TE5	www.whoopingcough.net/images/whooping%20cough%2030%20second%20mpg.mpg	Pertussis	Child	30
TE6	www.whoopingcough.net/images/videochildwhoop3.wmv	Pertussis	Child	92
TE7	https://www.youtube.com/watch?v=VX98aiYpmW4	Pertussis	Infant	106
TE8	https://www.youtube.com/watch?v=yv4GUrI0Cw4	Pertussis	Child	37
TE9	https://www.youtube.com/watch?v=zuK4honWVsE	Pertussis	Infant	26
TE10	https://www.youtube.com/watch?v=PFNvGqw9HKY	Pertussis	Child	13
TE11	https://www.youtube.com/watch?v=_vgOOuBKKu8	Croup	Child	58
TE12	https://www.youtube.com/watch?v=3eJQAdkW1Aw	Unknown NP	Child	62
TE13	https://www.youtube.com/watch?v=iQit0aZ_Sbg	Unknown NP	Child	15
TE14	https://www.youtube.com/watch?v=fWUoarRzAwY	Unknown NP	Child	42
TE15	https://www.youtube.com/watch?v=gus1GHeS7IE	Unknown NP	Infant	16
TE16	https://www.youtube.com/watch?v=IYllzXfvkmY	Bronchitis	Adult	53
TE17	https://www.youtube.com/watch?v=IE_6K-ZfI64	Bronchiolitis	Infant	39
TE18	https://www.youtube.com/watch?v=GlzCDxaBB6w	Croup	Infant	12
TE19	https://www.youtube.com/watch?v=ooohsSgm5GM	Asthma	Adult	33
TE20	https://www.youtube.com/watch?v=5kAWlNZ-I_I	Bronchiolitis	Infant	44
TE21	https://www.youtube.com/watch?v=SsxsiISkLZA	Bronchiolitis	Infant	15

TR*x*—Training data; TE*x*—Test data; NP—Non-pertussis.

The first step for automated pertussis identification involves feature extraction from non-silent parts of an audio recording. Several features are extracted from each frame including time-domain features, frequency-domain features and Mel Frequency Cepstral Coefficients (MFCCs). These are subsequently used for cough detection, cough classification and whooping sound detection. The design and functionality of each block in the proposed algorithm is explained in the following sections.

### Sound Event Detection

**Fig 2:**
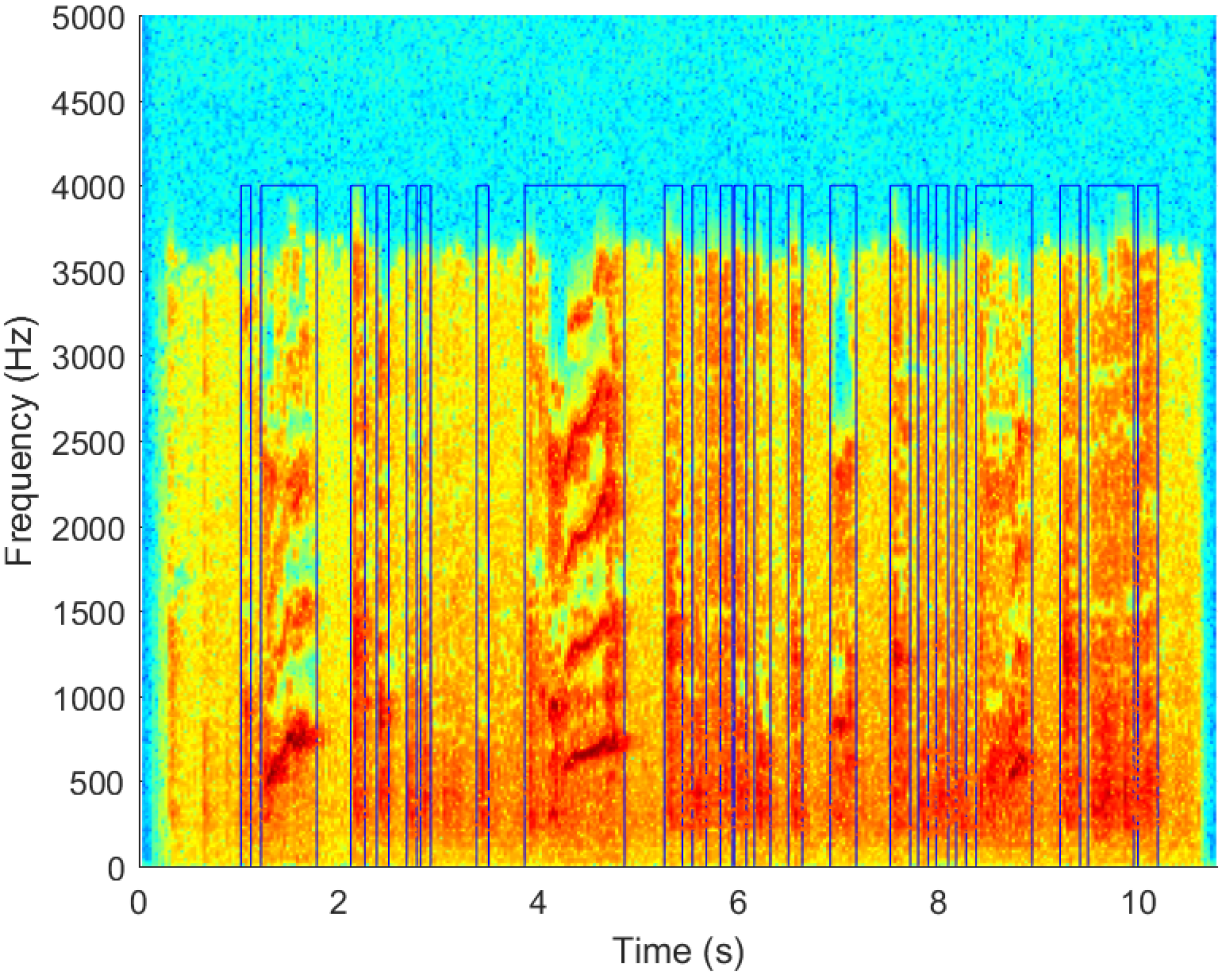
An example showing the output of sound event detection scheme where the non-silent parts of the recording have been successfully identified (areas under blue lines).

Prior to the detection of any cough and whooping events, a sound detector is used to remove the silent sections of the audio recordings. This ensures that all further audio processing is performed only on signals where there is some sound and also helps in reducing the processing load and decreases the algorithm runtime. It is implemented by comparing the standard deviation of each frame to the mean of the standard deviation in each recording. By setting a threshold for the minimum frame standard deviation the silent parts in a recording can be removed. An example of the result this approach achieves can be seen in Fig 2 where the sound events can be clearly distinguished from the silent parts of the recording.

### Feature Extraction

At the initial stages of algorithm development, a large number of features were extracted from the audio signals to study their discriminating abilities. From this initial set of features, that are explained below, the top performing ones were selected based on their usefulness in detecting and classifying cough and whoop sounds separately.

#### Mel-Frequency Cepstral Coefficient

The complex cepstrum is defined as the Fourier transformed logarithm of the signal spectrum [18]. The coefficients that represent this transformation are called cepstral coefficients *c*[*n*] and can be obtained using Eq (1) for a signal with power spectrum *S*(*ω*).

$$\begin{array}{c} log\left(S\left(\omega \right)\right)=\sum_{n=-\infty }^{\infty }c\left[n\right]e^{-jn\omega } \end{array}$$ (1)

A total of 13 MFCCs were extracted for the proposed algorithm, including the zeroth order MFCC, using the melcepst function in VOICEBOX speech processing toolbox [19] for Matlab [20]. Additionally, the first and second derivative coefficients were also extracted, such that there were a total of 39 MFCC features.

#### Zero-crossing Rate

Zero-crossing rate represents the frequency of sign-changes in a signal [21] and is calculated using Eq (2). It results in higher values for noisy and high frequency signals where sign changes are more frequent.

$$\begin{array}{c} ZCR=\frac{1}{T-1}\sum_{t=1}^{T-1}I\left\{s_{t}s_{t-1}<0\right\} \end{array}$$ (2)

#### Crest Factor

Crest factor is the ratio of the value of a peak in a waveform relative to its root mean square (RMS) value. Calculated using Eq (3), it gives a measure of the intensity of detected peaks.

$$\begin{array}{c} CF=\frac{{\left|x\right|}_{peak}}{x_{rms}} \end{array}$$ (3)

#### Energy Level

Energy level of an audio frame is calculated as the RMS of the frame using Eq (4). Since cough is an explosive sound, it will have bursts of energy increase in short time.

$$\begin{array}{c} Energy_{Level}=\sqrt{\frac{\sum_{n=0}^{N-1}x{\left(n\right)}^{2}}{N}} \end{array}$$ (4)

#### Dominant/Maximum Frequency (MaxF)

Dominant/maximum frequency is the value of the frequency bin at which the maximum power of the signal is found. Since the whooping sound has a significantly higher dominant frequency compared to the cough sound because of its higher pitch, this feature can be useful to distinguish between these two types of sounds.

#### Spectral Roll-Off (SRO)

Spectral roll-off determines the point below which most energy of a signal is contained and is useful in distinguishing sounds with different energy distributions. Both the cough and whooping sounds have different spectral roll-off frequencies since most energy in the cough sound is concentrated in the earlier sections of the frequency spectrum.

#### Spectral Skewness / Asymmetric Coefficient (SAC)

Spectral skewness or asymmetric coefficient is a measure of the asymmetry in the power spectrum of a signal about its mean (*μ*) and is useful in understanding if the power distribution in PSD estimate will have more density in lower or higher frequency. A negative skewness coefficient means that the distribution of the spectrum is left-tailed, while a positive coefficient signifies a right-tailed distribution. SAC is computed as the third moment divided by the cube of the standard deviation, as shown in the equation below.

$$\begin{array}{c} SAC={\left(\frac{E(x-\mu )}{\delta }\right)}^{3} \end{array}$$ (5)

#### Spectral Kurtosis Coefficient (SKC)

Spectral kurtosis coefficient is also a measure of the peak of the power spectrum and, similar to SAC, it can be used to describe the shape of the probability distribution of the energy in the power spectrum of a signal. A high kurtosis denotes more extreme infrequent deviation. From this feature, the power distribution peak, shoulder, and tail of the PSD estimate can be measured. SKC is computed by using the fourth moment as shown in Eq (6).

$$\begin{array}{c} SKC={\left(\frac{E(x-\mu )}{\delta }\right)}^{4} \end{array}$$ (6)

#### Spectral Centroid (SC)

Spectral centroid represents the equivalent of the center of mass in a spectrum and is computed as a weighted mean of the spectrum as shown in Eq (7) where *f*(*n*) is the frequency bin and while *x*(*n*) is the PSD estimate.

$$\begin{array}{c} SC=\frac{\sum_{n=0}^{N-1}f\left(n\right)x\left(n\right)}{\sum_{n=0}^{N-1}x\left(n\right)}=\mu_{1} \end{array}$$ (7)

#### Spectral Spread (SSp)

Spectral spread is a measure of the spread of a spectrum with respect to its mean. Also called spectral width, it is computed using Eq (8) where the moment *μ* is defined using Eq (9) [22].

$$\begin{array}{c} SS=\sqrt{\mu_{2}-SC^{2}}=\sqrt{\mu_{2}-\mu_{1}^{2}} \end{array}$$ (8)

$$\begin{array}{c} \mu_{i}=\frac{\sum_{n=0}^{N-1}f{\left(n\right)}^{i}x\left(n\right)}{\sum_{n=0}^{N-1}x\left(n\right)} \end{array}$$ (9)

#### Spectral Decrease (SD)

Spectral decrease represents the rate of spectral decrease and is calculated as follows.

$$\begin{array}{c} SD=\frac{1}{\sum_{n=2}^{N}x}\sum_{n=2}^{N}\frac{x(n)-x(1)}{n-1} \end{array}$$ (10)

#### Spectral Flatness (SF)

Spectral flatness determines the flatness of a spectrum by comparing its geometric with the arithmetic mean. Also called tonality coefficient and calculated using Eq (11), it helps to quantify how noise-like a sound is.

$$\begin{array}{c} SF=\frac{e^{\left(\frac{1}{N}\sum_{n}log\left(x\left(n\right)\right)\right)}}{\frac{1}{N}\sum_{n}x\left(n\right)} \end{array}$$ (11)

#### Spectral Slope (SSl)

Spectral slope measures the decreasing slope of a spectrum and indicates how quickly the power of a spectrum goes down towards high frequencies. Based on [23], it can be computed by linear regression of the spectrum as shown below.

$$\begin{array}{c} SSl=\frac{1}{\sum_{n}x\left(n\right)}\frac{N\sum_{n}f\left(n\right)x\left(n\right)-\sum_{n}f\left(n\right)\sum_{n}x\left(n\right)}{N\sum_{n}f^{2}\left(n\right)-{\left(\sum_{n}f\left(n\right)\right)}^{2}} \end{array}$$ (12)

#### Spectral Standard Deviation (SSD)

Spectral standard deviation is a commonly used feature that measures the standard deviation of the PSD.

#### Band Power (BP)

Band power represents the average power in a specific frequency band. It is calculated by integrating the PSD estimate using rectangle approximation method.

### Cough detection

It is important to understand the characteristics of a cough sound before any attempt of its detection is made. A cough sequence is started by a mechanical or chemical stimuli and is ended when the unwanted substances are removed from the airways [24]. The acoustic features of a cough sound depends on the airflow velocity as well as the dimensions of vocal tract and airways [24]. This makes it possible to detect or classify a cough sound based on the acoustic features since these features are dependent on the cause of cough.

In order to perform cough detection from the audio signal, the features described in the earlier section are used as predictors in a logistic regression model (LRM). This cough sound detection model is trained using 10 pertussis and 7 non-pertussis recordings from the database. The cough sounds in each recording are manually segmented and clearly marked to allow for binary classification. The top nine features are then selected using sequential feature selection since addition of further features result in very small changes to the model deviance. The final list of features used for this classifier is shown in Table 2 in order of maximum deviance minimization.

**Table 2:** List of features used for cough sound detection.

No.	Feature
1	MFCC
2	Crest Factor
3	Spectral Flatness
4	Band Power
5	Spectral Roll Off
6	Max Frequency
7	Spectral Standard Deviation
8	Spectral Kurtosis
9	Spectral Slope

### Cough classification

Chung et al. [25] classified cough sounds based on five different categories: behavioral, pathological, duration, effect and grade of coughs. Whooping cough, which is the focus of this work, is considered to be a pathological cough. The types of cough based on pathology, aside from whooping cough, are dry cough, wet cough, hacking, throat or chest irritation and nasal drip. However, a whooping cough is basically a series of dry coughs followed by whooping sound making it somewhat similar to the coughs in other conditions. Thus, it is important to clearly distinguish whooping cough from other conditions with dry cough as a symptom, such as croup or bronchiolitis.

From observing a cough sound signal in time domain Morice et al. [26] concluded that there are three types of cough patterns. This includes 3-phased cough (which is the most common type of cough sound signal) [27], 2-phased cough, and peal cough. For a 3-phased cough, Korpas et al. [28] concluded that the first phase of the cough is due to turbulent airflow which itself is caused by narrowed airways. This leads to vibrations in the airway as well as the lung tissue. In case of whooping cough, the airways will be filled with a lot of thick mucus which may cause stronger vibrations as the airways become narrower. Subsequently, this may lead to higher power during the first phase of the cough. The second phase of the cough is caused by the airflow in the trachea, while the final phase is induced by the adduction of the vocal fold at the end of the second phase [28].

The cough sounds detected are quite generic and may result from a number of different medical conditions. This section describes how the detected cough sounds are classified to determine if they are the kind of dry coughs that are specific to pertussis. A separate logistic regression model is used to perform the classification of the isolated cough events. From the dataset, half of the cough events are used to train the LRM and the other half are used for testing. The features extracted from the training set include all the time and frequency domain features listed earlier with 13 MFCCs including the zeroth coefficient. Each isolated cough event is divided into three same-length sections following the 3-phased cough model and a total of 30 features are extracted from each section. With these features, an LRM classifier is used to determine whether the isolated cough sounds are of the kind that is heard in pertussis or not. However, not all of the detected cough sounds are used for the automatic classification. Some of the extracted sound events have length that are not typical of a cough sound. Only sound events with length typical to a cough sound are selected to be used in the automatic classification while others are discarded by setting a threshold for duration. The final result of this classifier is the percentage of cough events classified as a pertussis case relative to the total number of coughs.

### Whooping sound detection

Although the whooping sound normally follows an episode of coughing, it is not necessarily present in all cases of pertussis nor in every spell of coughing, especially in the case of infants. However, in cases where this sound is present, its detection helps to improve the diagnosis of pertussis and improve the overall accuracy an automated classifier.

The design of the whooping sound detector follows a similar pattern to the cough detector. Of the 38 recordings in the database, 10 pertussis and 7 non-pertussis recordings are used to create the training set. The MFCCs, time and frequency domain features listed earlier are extracted from these recordings to create a feature vector for a logistic regression model.

For feature selection, the features are added one by one to minimize the model deviance at each step. Once the reduction in deviance becomes very small with each additional feature, this process is stopped and only the top 12 features are used. This ensures the use of minimum number of features to achieve the highest classification performance. Table 3 lists the features used in order of maximum deviance minimization.

**Table 3:** List of features used for whooping sound detection.

No.	Feature
1	MFCC
2	Spectral Standard Deviation
3	Crest Factor
4	Spectral Spread
5	Spectral Skewness
6	Spectral Flatness
7	Spectral Roll Off
8	Zero Crossing Rate
9	Band Power
10	Spectral Slope
11	Spectral Kurtosis
12	Max Frequency

### Pertussis Diagnosis

The results from cough detection followed by classification and whooping sound detection are collated in order to provide the final pertussis identification. If a whooping sound is detected in an audio recording, then the case is identified as a pertussis even if the pertussis cough ratio is low. If there is no whooping sound detected, the identification result is obtained from the pertussis cough ratio obtained from the cough classifier. Without the whooping sound, if the pertussis cough ratio is greater than 0.5 then the case is classified as pertussis.

## Results

There are three different instances of classification being performed in the algorithm before the final pertussis diagnosis is determined. The first one is the identification of individual cough instances to determine whether an audio sound is in fact a cough sound or not. Once this is complete, the next stage involves classification of these cough sounds. A parallel classifier attempts to identify the presence of whooping sounds. These results are then used to mark a recording as either pertussis or non-pertussis.

In this section the results of all these classifiers are presented individually to assess their performance on both training and test data sets using the following metrics [29].

1. **Sensitivity**, which represents the fraction of correctly identified positive cases.
2. **Specificity**, which represents the fraction of negatives cases being correctly rejected.
3. **Positive Predictive Value (PPV)**, which represents the proportion of positive results that are correctly detected.
4. **Negative Predictive Value (NPV)**, which represents the proportion of negative results that are correctly rejected.

To calculate the metrics above, *TP* (True Positives) is the number of instances correctly detected as either cough or whooping sound (depending on the classifier), *FP* (False Positives) is the number of incorrectly scored instances, *TN* (True Negatives) is the number of instances correctly rejected, and *FN* (False Negatives) is the number of incorrectly rejected instances. For pertussis diagnosis, the PPV and NPV are the most important metrics since they indicate the degree of confidence with which a diagnosis is made.

### Cough detection

**Fig 3:**
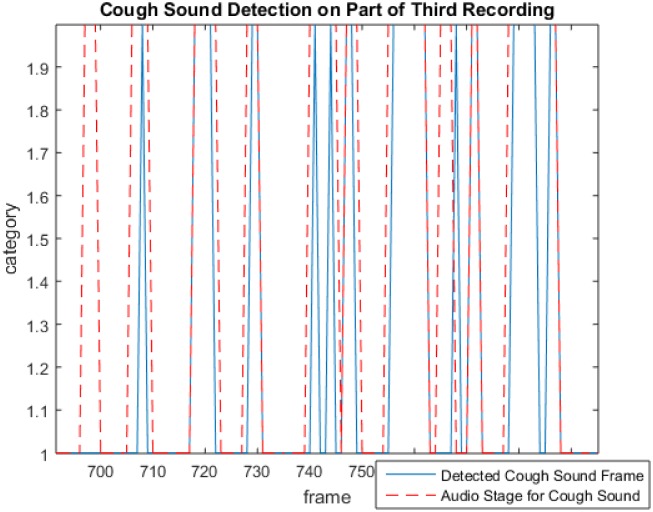
An example illustrating the output of cough detection with red lines showing the reference cough frames and blue lines showing the detected cough frames.

Fig 3 shows an example of several cough instances being detected from an audio recording. The dotted lines show the reference cough frames in the recording while the solid lines show the frames classified as cough sound by the logistic regression model. In this example there are a total of ten actual cough events of which nine are detected (true positives) while one goes undetected and is counted as a false negative.

Table 4 shows the overall cough detection performance across all test recordings as well as the individual performance for each of the 21 recordings. It shows the total coughs present in each audio recording of the test dataset and the fraction of correctly detected coughs. Of the total 414 cough events across 21 recordings, 85% of them are correctly detected with a combined PPV of 85%. In most cases with pertussis the sensitivity values are more than 80% individually with an average of 89% except in case 2 where the sensitivity is 65%. This is because of the recording consists of other people speaking at the time of cough events making it difficult to detect the cough sounds individually. However, the high specificity and low number of false positives indicate that the classifier is still able to reject other sounds which are not cough with a high accuracy. Further. in non-pertussis cases (11-21), the sensitivity values are generally lower. This is due to the lower number of reference cough sounds resulting in greater changes in overall sensitivity even when a smaller number of coughs are not detected.

**Table 4:** Performance of the algorithm for cough sound detection using test data.

Case	Diag	TP	TN	FP	FN	Sen (%)	Spe (%)	PPV(%)	NPV(%)
1	P	59	342	4	4	93.65	98.84	93.65	98.84
2	P	36	125	3	19	65.45	97.66	92.31	86.81
3	P	15	45	1	0	100.00	97.83	93.75	100.00
4	P	6	86	7	0	100.00	92.47	46.15	100.00
5	P	13	131	1	3	81.25	99.24	92.86	97.76
6	P	67	302	8	0	100.00	97.42	89.33	100.00
7	P	21	544	19	1	95.45	96.63	52.50	99.82
8	P	15	165	0	10	60.00	100.00	100.00	94.29
9	P	17	87	0	0	100.00	100.00	100.00	100.00
10	P	12	41	2	1	92.31	95.35	85.71	97.62
11	NP	1	335	0	5	16.67	100.00	100.00	98.53
12	NP	7	354	5	1	87.50	98.61	58.33	99.72
13	NP	4	72	1	2	66.67	98.63	80.00	97.30
14	NP	1	247	0	3	25.00	100.00	100.00	98.80
15	NP	8	67	1	0	100.00	98.53	88.89	100.00
16	NP	7	208	1	5	58.33	99.52	87.50	97.65
17	NP	12	162	8	1	92.31	95.29	60.00	99.39
18	NP	5	54	0	0	100.00	100.00	100.00	100.00
19	NP	13	152	0	1	92.86	100.00	100.00	99.35
20	NP	21	176	3	2	91.30	98.32	87.50	98.88
21	NP	12	40	0	4	75.00	100.00	100.00	90.91
Total		352	3735	64	62	85.02	98.32	84.62	98.37

Diag—indicates whether recording has pertussis (P) or non-pertussis (NP) diagnosis. TP—true positives; TN—true negatives; FP—false positives; FN—false negatives. Sen—sensitivity; Spe—specificity; PPV—positive predictive value; NPV—negative predictive value.

### Cough classification

Once the cough sounds are detected, they are checked to see if they are similar to the cough sound that is generally observed in pertussis. For this, the classifier is trained using cough sounds that are manually isolated from the audio recordings. Half of the manually segmented cough sounds are used for training the model and the other half for testing. The average performance achieved for the cough sound classification into either pertussis or non-pertussis cough is shown in Table 5. All of the metrics indicate a good classification performance except NPV which is slightly lower at 80%. This is perhaps due to the presence of more pertussis cough sounds in the database.

**Table 5:** Performance of the algorithm for cough classification using test data.

Metric	Value (%)
Sensitivity	92.38
Specificity	90.00
PPV	96.50
NPV	79.84

Table 6 shows that while the NPV is high for infants, it is much lower for children. For this age agroup, the PPV is alse low despite a high sensitivity. This represents an area where more work is needed to improve the classifier performance. It is possible to explore age-dependent features in order to achieve higher accuracy.

**Table 6:** Cough classification performance by age group.

	Sensitivity (%)	Specificity (%)	PPV (%)	NPV (%)
Infants	92.59	92.86	94.94	89.66
Children	93.00	70	69.88	50.00
Adults	89.36	92.31	97.67	70.59
Overall	92.38	90.00	96.50	79.84

The performance of this cough classifier is also assessed in combination with the cough detection block which automatically detects the individual cough instances. The number of coughs, for each test case, that are classified as pertussis cough as a fraction of the total number of detected coughs is then computed. This is a number between 0 and 1 that gives a fair probability of pertussis diagnosis based on a large number of coughs for an individual. These results are shown in Table 7 which clearly demonstrates a significantly higher percentage of pertussis coughs being classified in cases which are already diagnosed with pertussis.

**Table 7:** Performance of the algorithm for cough classification together with cough sound detection using test data.

Case	Diag	Pertussis Cough Ratio
1	P	0.98
2	P	0.70
3	P	1.00
4	P	0.67
5	P	0.91
6	P	1.00
7	P	0.90
8	P	1.00
9	P	0.93
10	P	1.00
11	NP	0.00
12	NP	0.50
13	NP	0.00
14	NP	0.00
15	NP	0.22
16	NP	0.00
17	NP	0.46
18	NP	0.25
19	NP	0.25
20	NP	0.00
21	NP	0.33

Diag—indicates whether recording has pertussis (P) or non-pertussis (NP) diagnosis.

### Whooping sound detection

**Fig 4:**
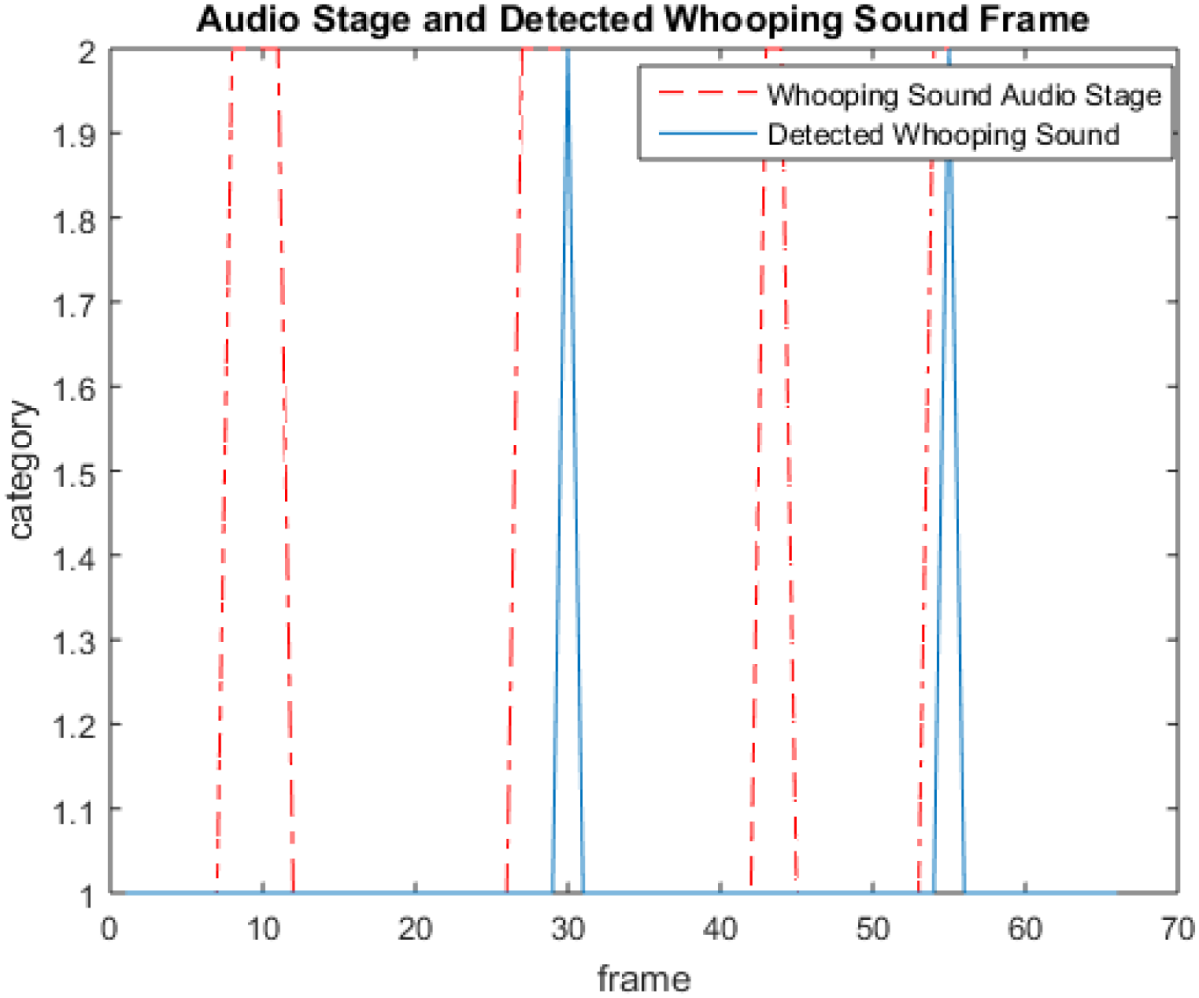
An example illustrating the output of whooping sound detection with red lines showing the reference whooping sound frames and blue lines showing the detected whooping sound frames.

Fig 4 shows an example of whooping sound detection being performed where the dashed lines represent the reference whooping sound while the solid lines show the whooping sound as detected by the algorithm. In this example, there are four frames with whooping sound of which two are correctly detected by the algorithm and two are incorrectly rejected.

While the example shows a clear case of pertussis with the presence of whooping sound, there are some recordings in the test dataset lacking this whooping sound despite being diagnosed as pertussis. Further, no whooping sound is present in the recordings of non-pertussis cases. The complete results of the whooping sound detector for recordings from the test data set are shown in Table 8. The overall sensitivity in this case is 73% with the PPV value of 87%. In pertussis cases, there are two recordings without the presence of any whooping sound and both of these result in no false positives being detected by the classifier. Of the remaining eight, at least some of the whooping sound segment gets detected in six cases. In non-pertussis cases, there is no false detection of whooping sound resulting in specificity of 100% in all cases.

**Table 8:** Performance of the algorithm for whooping sound detection using test data.

Case	Diag	Whoop	TP	TN	FP	FN	Sen (%)	Spe (%)	PPV(%)	NPV(%)
1	P	Y	1	528	0	0	100.00	100.00	100.00	100.00
2	P	Y	15	234	2	0	100.00	99.15	88.24	100.00
3	P	N	0	99	0	0	-	100.00	-	100.00
4	P	Y	1	113	0	2	33.33	100.00	100.00	98.26
5	P	Y	4	165	0	0	100.00	100.00	100.00	100.00
6	P	Y	1	565	0	1	50.00	100.00	100.00	99.82
7	P	Y	0	640	0	5	0.00	100.00	-	99.22
8	P	Y	0	229	0	1	0.00	100.00	-	99.57
9	P	Y	5	127	2	1	83.33	98.45	71.43	99.22
10	P	N	0	82	0	0	-	100.00	-	100.00
11	NP	N	0	364	0	0	-	100.00	-	100.00
12	NP	N	0	387	0	0	-	100.00	-	100.00
13	NP	N	0	95	0	0	-	100.00	-	100.00
14	NP	N	0	265	0	0	-	100.00	-	100.00
15	NP	N	0	102	0	0	-	100.00	-	100.00
16	NP	N	0	332	0	0	-	100.00	-	100.00
17	NP	N	0	248	0	0	-	100.00	-	100.00
18	NP	N	0	75	0	0	-	100.00	-	100.00
19	NP	N	0	207	0	0	-	100.00	-	100.00
20	NP	N	0	275	0	0	-	100.00	-	100.00
21	NP	N	0	95	0	0	-	100.00	-	100.00
Total			27	5227	4	10	72.97	99.92	87.10	99.81

Diag—indicates whether recording has pertussis (P) or non-pertussis (NP) diagnosis. Whoop—indicates whether recording has whooping sound; Y—Yes; N- No. TP—true positives; TN—true negatives; FP—false positives; FN—false negatives. Sen—sensitivity; Spe—specificity; PPV—positive predictive value; NPV—negative predictive value.

### Pertussis Diagnosis

The complete pertussis identification results for the test data are shown in Table 9. From the table, it can be seen that all cases have been successfully identified as either pertussis or non-pertussis. In cases 1-10 (with pertussis diagnosis), six cases have been identified because the presence of whooping sounds have been detected. In the other four, the pertussis cough ratio is greater than 0.5 indicating a high likelihood of pertussis. For non-pertussis cases, the cough ratio is either zero or very low for all except case 12 which is on the border. The identification threshold can be increased if a more strict detection criterion is desired.

**Table 9:** Performance of the algorithm for pertussis diagnosis using test data.

Case	Diag	Whooping Sound	Pertussis Cough Ratio	Pertussis Identification
1	P	1	0.98	P
2	P	1	0.70	P
3	P	0	1.00	P
4	P	1	0.67	P
5	P	1	0.91	P
6	P	1	1.00	P
7	P	0	0.90	P
8	P	0	1.00	P
9	P	1	0.93	P
10	P	0	1.00	P
11	NP	0	0.00	NP
12	NP	0	0.50	NP
13	NP	0	0.00	NP
14	NP	0	0.00	NP
15	NP	0	0.22	NP
16	NP	0	0.00	NP
17	NP	0	0.46	NP
18	NP	0	0.25	NP
19	NP	0	0.25	NP
20	NP	0	0.00	NP
21	NP	0	0.33	NP

Diag—indicates whether recording has pertussis (P) or non-pertussis (NP) diagnosis.

## Discussion

In this paper, an algorithm for automated pertussis diagnosis is presented with additional identification of other diagnostic features. The algorithm consists of LRM-based classifiers for whooping sound detection, cough sound detection, and cough sound classification. This algorithm represents the very first reported attempt towards a fully automated end-to-end solution that incorporates automatic cough detection and classification as well as whooping sound detection. As a result, no manual segmentation of cough sounds need to be performed allowing the algorithm to be used as a stand-alone solution in real-time. Additionally, it does not require any person-specific tuning of thresholds, is computationally efficient and is resilient to artifacts enabling unsupervised usage on smartphones under real-world conditions. The main contribution of the work presented in this paper is a complete pertussis diagnosis algorithm, however, the intermediate classifiers for cough detection, classification and whooping sound detection can be used on their own for various applications.

The cough detection part of the diagnosis algorithm presented here, if used on its own, achieves performance that is comparable to other methods proposed in literature. This is despite its lower complexity compared to other cough detection methods [8, 10, 11, 30] that use HMM, SVM and neural networks for classification.

Algorithms for cough classification have also been published previously including those for pneumonia [16, 31, 32], wet and dry cough classification [14, 15, 33] and asthma [34]. However, the only other study for pertussis cough classification is published by Parker et al. [17]. This uses neural networks to classify coughs with sensitivity and specificity of 93% and 92% respectively for 47 cough events only. In comparison, the cough classifier part of the diagnosis algorithm proposed in this paper uses significantly more cough events as part of the test data and achieves performance comparable to that in [17]. Its performance can be improved further by incorporating other types of coughs such as those in croup, bronchiolitis, asthma, and cold cough. Additionally, the proposed cough classifier also computes the pertussis cough ratio that minimizes the effects of misclassification at the final pertussis identification stage.

The whooping sound detection part of the diagnosis algorithm, when used independently, has a very high specificity but is limited by its lower sensitivity. It should be noted that whooping sound is categorized as a *pathognomonic* symptom for pertussis. This means that whooping sound is a unique characteristic of pertussis. By developing a high specificity detector, a pertussis case can be objectively confirmed by the presence of a whooping sound.

The pertussis diagnosis algorithm proposed in this paper successfully identifies all the cases correctly resulting in 100% accuracy. However, it has been tested on a limited amount of test data consisting of 10 pertussis and 11 non-pertussis audio recordings. Further, the laboratory confirmation methods to obtain diagnosis in this data are not known. This is a limitation of the current preliminary study and further work is needed for validation with more data and known lab classification methods. If needed, the algorithm performance can also be enhanced by exploring the use of more features and improved intermediate classifiers. However, the use of more complex classifiers, such as neural networks, comes at the cost of added computational complexity. Depending on where the algorithm is to be implemented, certain feature extraction and classification methods can be computationally prohibitive. Additionally, when implemented on a smartphone, a series of questions can be asked to obtain user input which can supplement the decision-making process for the diagnosis. These may include questions about the immunization status of an individual, clinical symptoms that vary by age, lifestyle-related information e.g. smoking habits, and questions about related prior problems.

While the main aim of the work presented in this paper is to target low-resource areas for diagnosing pertussis where clinical facilities are limited, it has other clinical and educational applications as well. It can be very useful to demonstrate and teach students the differences between various kinds of cough sounds. Further, it can be used for differential diagnosis of respiratory infections where symptoms of illnesses are similar. For example, pertussis cases may be misdiagnosed as bronchiolitis with further impacts such as missed antibiotic treatments. The use of our proposed method allows for the possibility of distinguishing between these two despite their similar symptoms otherwise.

Overall, the algorithm proposed in this paper achieves a high pertussis identification performance with simple classification methods. This shows that a pertussis cough can be automatically identified using its sound characteristics with a high degree of confidence and can be implemented on mid-range smartphones. This is particularly important in case of a whooping cough outbreak in locations where sophisticated laboratory tests and specialists may not be available. When implemented on a portable device, such as a smartphone or tablet, the algorithm can be extremely useful for quick identification or early screening of pertussis and for infection outbreaks control.
